# Image quality improvement using model-based iterative reconstruction in low dose chest CT for children with necrotizing pneumonia

**DOI:** 10.1186/s12880-017-0177-9

**Published:** 2017-03-16

**Authors:** Jihang Sun, Tong Yu, Jinrong Liu, Xiaomin Duan, Di Hu, Yong liu, Yun Peng

**Affiliations:** 1 Imaging Center, Beijing Children’s Hospital, Capital Medical University, No.56, Nanlishi Road, Xicheng District, Beijing, 100045 People’s Republic of China; 2 Department of respiratory, Beijing Children’s Hospital, Capital Medical University, Beijing, 100045 People’s Republic of China

**Keywords:** Computed tomography (CT), Model-based iterative reconstruction, Filtered back-projection, Necrotizing pneumonia, Child

## Abstract

**Background:**

Model-based iterative reconstruction (MBIR) is a promising reconstruction method which could improve CT image quality with low radiation dose. The purpose of this study was to demonstrate the advantage of using MBIR for noise reduction and image quality improvement in low dose chest CT for children with necrotizing pneumonia, over the adaptive statistical iterative reconstruction (ASIR) and conventional filtered back-projection (FBP) technique.

**Methods:**

Twenty-six children with necrotizing pneumonia (aged 2 months to 11 years) who underwent standard of care low dose CT scans were included. Thinner-slice (0.625 mm) images were retrospectively reconstructed using MBIR, ASIR and conventional FBP techniques. Image noise and signal-to-noise ratio (SNR) for these thin-slice images were measured and statistically analyzed using ANOVA. Two radiologists independently analyzed the image quality for detecting necrotic lesions, and results were compared using a Friedman’s test.

**Results:**

Radiation dose for the overall patient population was 0.59 mSv. There was a significant improvement in the high-density and low-contrast resolution of the MBIR reconstruction resulting in more detection and better identification of necrotic lesions (38 lesions in 0.625 mm MBIR images vs. 29 lesions in 0.625 mm FBP images). The subjective display scores (mean ± standard deviation) for the detection of necrotic lesions were 5.0 ± 0.0, 2.8 ± 0.4 and 2.5 ± 0.5 with MBIR, ASIR and FBP reconstruction, respectively, and the respective objective image noise was 13.9 ± 4.0HU, 24.9 ± 6.6HU and 33.8 ± 8.7HU. The image noise decreased by 58.9 and 26.3% in MBIR images as compared to FBP and ASIR images. Additionally, the SNR of MBIR images was significantly higher than FBP images and ASIR images.

**Conclusions:**

The quality of chest CT images obtained by MBIR in children with necrotizing pneumonia was significantly improved by the MBIR technique as compared to the ASIR and FBP reconstruction, to provide a more confident and accurate diagnosis for necrotizing pneumonia.

## Background

With the increased demand for CT, the desire to reduce radiation dose also increases. This is especially true in pediatric CT imaging. However, the conventional filtered back-projection (FBP) reconstruction limits the possibility of further reducing the radiation dosage, especially when higher spatial resolution and thinner image slice thickness are desirable. Hence, the intention of reducing the radiation dosage in CT has in recent years shifted focus from hardware upgrades and tube current decrease only to the development of advanced reconstruction algorithms. Noise model-based iterative reconstruction, such as adaptive statistical iterative reconstruction (ASIR), iDose and Sinogram affirmed iterative reconstruction (SAFIRE) [1–5], is a new type of CT reconstruction algorithm using statistical models to reduce image noise and produce better image quality [6, 7]. These new iterative reconstruction techniques have been applied in clinical practice and have demonstrated the ability to provide clinically acceptable images for diagnosis with significantly reduced radiation dosage by 32–65% [7].

Recently, a full model-based iterative reconstruction (MBIR) algorithm that incorporates both the noise mode and the optical model of the whole detection system for significantly decreasing image noise and improving spatial resolution at the same time has been introduced. MBIR algorithm has been used in adults for reducing image noise and radiation dose in chest and lung field [8–12], but few studies have been done in children [13, 14], and there is no research about the effect of iterative algorithm on image quality and dose in children with necrotic lesions. The purpose of this study was to evaluate the improvements in chest CT image quality and diagnostic performance with MBIR over FBP and ASIR in children with necrotizing pneumonia (NP) in simulated extremely low signal (radiation dose) CT scans.

## Methods

### Patient population

This retrospective study was approved by the Ethics Committees of Beijing Children’s Hospital for using the data, and parent consent was waived. One hundred and forty-one children with suspected NP, congenital lung diseases, trachea malformation or vessel malformation underwent low dose enhanced chest CT scans in our hospital between March 11, 2012 and August 31, 2013. Of these, 26 children (aged 2 months to 11 years with a median age of 4.0 years) were clinically diagnosed for NP. There were 3 patients in the 0–12 month age group, 5 in the 1–2 years and 18 in the 3–17 years age groups. Among these, 15 children had *Mycoplasma pneumoniae* (combined with *Klebsiella pneumonia* infection in one patient), two children had group A beta-hemolytic *Streptococci*, two children had *Streptococcus pneumonia* infections, and seven children had no clear etiological diagnosis. The time interval between the enhanced CT examination and the onset of the symptoms (fever, cough, or other symptoms) was 30.2 ± 11.1 days. In total, 33 necrotic lesions were observed in 28 pulmonary lobes in the standard CT images (5 mm thickness FBP image). Twenty of the lesions involved 17 lobes in the right lung (1 lesion in 1 upper lobe; 3 lesions in 3 middle lobes; 16 lesions in 13 lower lobes) and 13 lesions involved 11 lobes in the left lung (2 lesions in 2 upper lobes; 4 lesions in 3 middle lobes; 7 lesions in 6 lower lobes).

### Data acquisition

All patients underwent a contrast-enhanced low dose CT scan on a 64 rows high definition CT (HDCT) scanner (Discovery CT 750 HD, GE Healthcare, Waukesha, WI USA) with a tube voltage of 120 kVp, pitch of 1.375, detector collimation was 64x0.625 mm for a 40 mm total coverage in a single rotation, and rotation speed of 0.8 s. The tube current was set between 10 and 350 mA, and modulated with the automatic tube current modulation (ATCM) system. Age-dependent noise index (NI) settings for a 5 mm thick image were used for the acquisition: NI = 11 for 0–12month old, NI = 13 for 1–2 years old and NI = 15 for 3–17years old. Most children were too young to cooperate and were sedated with oral chloral hydrate (10%, 0.5 mL/kg) before scanning. The CT scan covered the area between the entrance to the chest and the base of the lungs. Iodinated contrast (Iodixanol 320, 320 mg I/mL, visipaque^TM^; GE Healthcare, Ireland, IDA Business Park Carrigtohill Co. Cork) was intravenously administered; contrast dose was based on body weight with 1.8, 1.6, 1.4, 1.2, and 1.0 mL/kg, for children weighing 3–5 kg, 5–10 kg, 10–15 kg, 15–35 kg, and >35 kg, respectively. Contrast was administered using a single-head power injector at injection rates of 0.4–3.0 mL/s adjusted to a fixed injection time of 15 s. Enhanced scans started at 45 s after the start of contrast injection.

### Subjective assessment of the image quality

The original image sets were reconstructed at 5 mm slice thickness with a filtered back-projection (FBP) algorithm according to the standard protocol for image review and diagnosis. The scan data were further retrospectively reconstructed into three groups with thinner slice thickness of 0.625 mm based on the reconstruction algorithm: series A with MBIR technique, series B with 30%ASIR (blending of 30% of ASIR and 70% of FBP reconstruction), which was recommended by vendor to have balanced image noise and spatial resolution, series C with FBP technique. The division of image slice thickness from 5 to 0.625 mm was used to simulate an extremely low signal but higher resolution study. All images were transferred to a GE AW4.5 CT workstation for analysis. The reviewers were blinded to background information about the children, scanning parameters and reconstruction algorithms. Two experienced radiologists (one with 13 years of experience in adult radiography, and 6 years’ experience in pediatric radiography, and the other one with 3 years of experience in adult radiography and 4 years of experience in pediatric radiography) evaluated the images independently. The objective image quality evaluation was performed on the three 0.625 mm slice thickness image sets (with MBIR, ASIR and FBP). The images were displayed initially in the mediastinal window, but the reviewers could adjust the window width and window level as per their preference. Multi-planar reconstruction (MPR) and Volume rendering (VR) images were also available for viewing. The thickness of MPR was also 0.625 mm.

Subjective display ability for necrotic lesions and confidence in diagnosis were used to rate image quality, with a 5-point scale. The ability for the necrotic lesions display was scored as follows: 5 = lesion(s) border displayed clearly with excellent contrast to surrounding tissue; 4 = lesion(s) margin displayed clearly with good contrast; 3 = lesion(s) displayed with marginal contrast, but a little blurry; 2 = lesion(s) margin displayed with poor contrast and incompletely; and 1 = cannot distinguish necrosis lesions from surrounding tissue. Diagnosis confidence was scored as, 5 = the whole image displayed perfectly with excellent confidence; 4 = the whole image has small amount of noise but with good confidence; 3 = there are some noise but image quality is good enough for diagnosis; 2 = whole image is not clear enough for confident diagnosis; and 1 = very hard to distinguish structures and cannot make any diagnosis.

### Evaluation of the objective noise

Quantitative image noise measurement was performed on all 4 image sets: both 5 and 0.625 mm slice thickness images with FBP reconstruction, 0.625 mm slice thickness with ASIR algorithm and 0.625 mm slice thickness with MBIR algorithm. After the qualitative evaluation, the two radiologists selected the largest cross-section area of the consolidated lesion (Le) and necrotic area (Nec) in the lesion and drew regions of interest (ROI) together in consensus. The CT density was measured in an area of ROI of 8–39 mm^2^, and the standard deviation (SD) was calculated. CT densities and SD of the back muscles (Mus) and background air (Air) in the same cross section were also measured. The objective image noise was represented using the average SD for these areas. Signal-to-noise ratio (SNR) for all tissues was calculated using the following formula: SNR = CT density_(ROI)_/SD_(ROI)_. Contrast noise ratio (CNR) for the consolidated lesion to necrotic area (CNR_(L-C)_) and consolidated lesion to air (CNR_(L-A)_) were calculated using the following formula: CNR_(L-C)_ = (CT density_(Le)_–CT density_(Nec)_)/SD_(muscle)_, CNR_(L-A)_ = (CT density_(Le)_–CT density_(Air)_)/SD_(Air)_ . The ROI was generally about 1 quarter of the cross-section area of the descending aorta at the same image layer, and the shape of the region was variable in the evaluation.

### Statistical analysis

The objective noise, SNR and CNR measurements and subjective image quality evaluation were recorded in detail and were presented as Mean ± SD. Objective measurements between MBIR, ASIR and FBP (0.625 and 5 mm) were compared statistically using one way ANOVA, To account for multiple statistical, a ‘Tukey’ post-hoc analysis was applied. And subjective image quality evaluation was compared using the Friedman’s test. Kappa statistics were used to evaluate the consistency between the two radiologists’ diagnoses. All statistical analyses were performed using SPSS17.0 (SPSS Inc., Chicago, IL, USA). A 2-tailed *P* value < 0.05 was deemed significant.

### Radiation dosage

Parameters of the X-ray radiation dosages including the volumetric CT dose index (CTDIvol) and dose length product (DLP) were recorded. Effective dose (ED) was calculated using the following formula: ED = DLP × W, where W is the patient age-dependent conversion factor for the chest area of pediatric patients. For our study, we used W values of 0.039, 0.026, and 0.018 for the patient age groups of 0–12 months, 1–2 years, and 3–6 years, respectively, based on the European guidelines on quality criteria for computed tomography to calculate an age-weighted conversion factor.

## Results

The CTDIvol, DLP, and overall radiation dose for the present study was 1.14 ± 0.56 mGy, 26.96 ± 9.86 mGy.cm, and 0.59 ± 0.19 mSv (using the age-weighted conversion factor of 0.022 mSv.mGy^−1^.cm^−1^ based on the age distribution of patient population in this study), respectively.

There were 33 necrotic lesions in 28 lobes detected in the standard 5 mm FBP images. In the 0.625 mm MBIR images, 38 necrotic lesions were found in the same 28 lobes; 3 necrotic lesions were missed in the 0.625 mm ASIR images, and only 29 necrotic lesions could be certain in the 0.625 mm FBP images due to high image noise.

### Subjective assessment of the image quality

The subjective image quality evaluation for the 3 thin image slice series are displayed in Table 1. MBIR images showed significantly better subjective image quality with significantly lower granular noise artifacts. There were significant differences between the 3 groups (*P* < 0.05).

**Table 1:** Subjective image quality evaluation among different reconstructions

Algorithm	Doctor A				Doctor B			
	Overall diagnosis confidence	*P*	Display ability for necrotic lesions	*P*	Overall diagnosis confidence	*P*	Display ability for necrotic lesions	*P*
MBIR	4.6 ± 0.5	-	5.0 ± 0.0	-	5.0 ± 0.0	-	5.0 ± 0.0	-
ASIR	2.9 ± 0.3	<*0.01*	2.6 ± 0.5	<*0.01*	3.0 ± 0.2	<*0.01*	3.0 ± 0.2	<*0.01*
FBP	2.7 ± 0.5	<*0.01*	2.3 ± 0.5	<*0.01*	2.9 ± 0.3	<*0.01*	2.6 ± 0.5	<*0.01*

### Objective image quality measurement

**Fig. 1:**
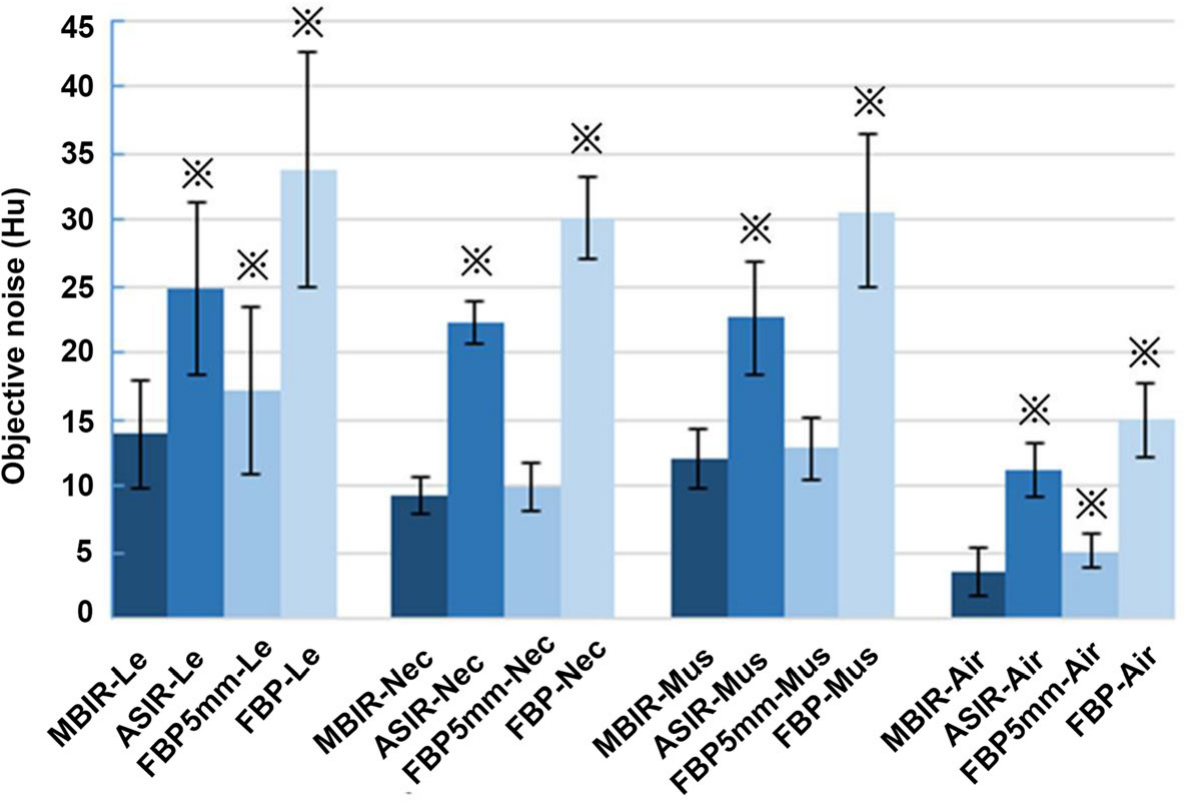
Average number and standard deviation of objective image noise of 4 algorithms on different tissues. Images were 0.625 mm in thickness unless stated otherwise. Le: consolidated lesion; Nec: necrotic area; Mus: muscle; Air: background air around body. ※ Significant difference of image noise compared with the 0.625 mm MBIR image

**Fig. 2:**
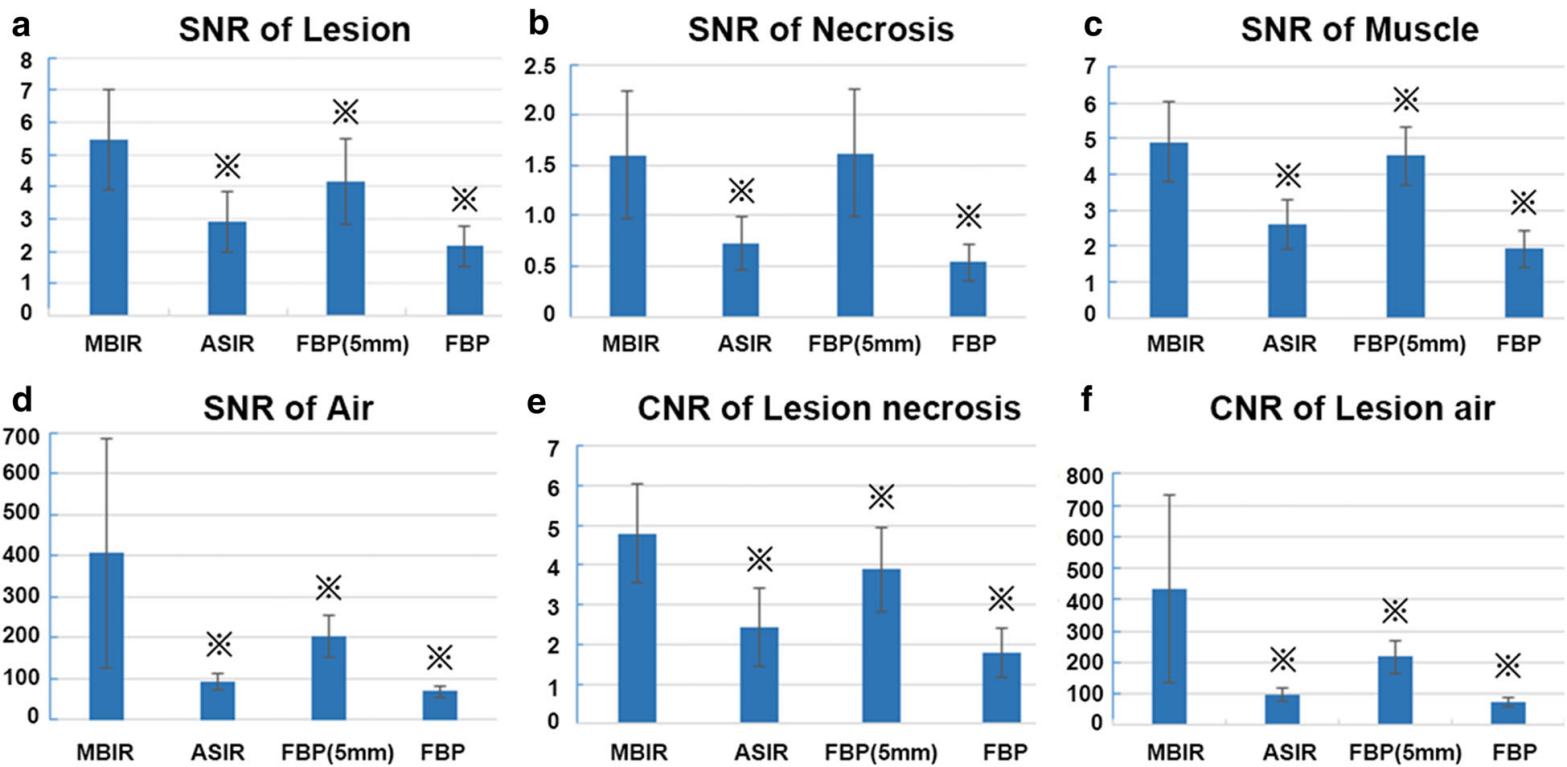
Signal-to-noise ratio (SNR) for the consolidated lesion (**a**); necrotic area (**b**); back muscles (**c**) and background air (**d**). Figures **e** and **f** show the contrast-to-noise ratio (CNR) for consolidated lesion contrast to necrotic area (**e**) and back muscles (**f**). SNR = CT density_(ROI)_/SD_(ROI)_. CNR_(E)_ = (CT density_(Lesion)_–CT density_(Necrosis)_)/SD_(muscle)_, CNR_(F)_ = (CT density_(Lesion)_–CT density_(Air)_)/SD_(Air)_. ※ : There is significant difference between MBIR image and other images

**Fig. 3:**
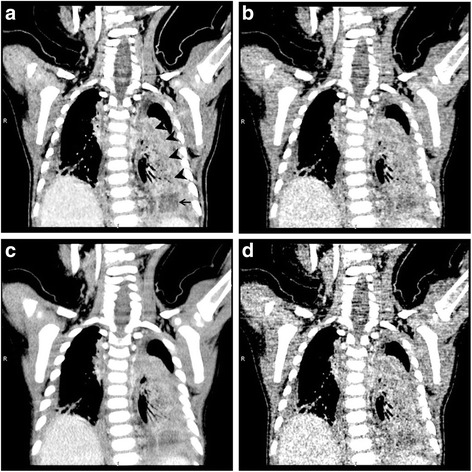
A child with group A beta-hemolytic streptococcus infection. CT scan was acquired at 120 kV with automatic tube current modulation technique (19–22 mA). Multi-planar reconstruction (MPR) was used for image review. (**a**, **b**, **c**, **d**) were MPR images with mediastinal window. **a** MBIR image with 0.625 mm; **b** ASIR image with 0.625 mm; **c** FBP image with 5 mm; **d** FBP image with 0.625 mm. Image noise of in MBIR image (**a**) was reduced significantly compared with that of 0.625 mm ASIR image (**b**) and 0.625 mm FBP image (**d**), and was similar to that of 5 mm FBP image (**c**). Necrotic lesions (*arrowheads*) and encapsulated pleural effusion (*arrow*) in the MBIR image (**a**) were displayed much clearly and confidently than in the 0.625 mm ASIR (**b**) and FBP (**d**) images. 5 mm FBP image (**c**) was too thick to display boundaries and scope of necrotic lesions

Figure 1 displays the objective image noise measurements; Fig. 2 displays the SNR and CNR results for all 4 image series. There were significant differences in the objective noise value among the 0.625 mm MBIR, ASIR and FBP images (*F* = 61.5, *P* < 0.05). There was significantly less objective noise for the consolidated lesions with the 0.625 mm thickness MBIR images than the thin slice ASIR and FBP images, and even the conventional 5 mm slice FBP images. The objective noise of consolidated lesions in the MBIR images was reduced by 44.2, 18.7 and 58.9%, compared with the 0.625 mm ASIR, 5 mm FBP and 0.625 mm FBP images, respectively (Fig. 3). Similarly, for the noise measurement in air, MBIR images reduced noise by a respective 67.9, 30.8 and 76.0%. The objective noise for the muscle in the 0.625 mm MBIR images was reduced by 46.7 and 60.6% compared to the 0.625 mm ASIR and FBP images, respectively, but image noise was statistically the same between the 0.625 mm MBIR and the 5 mm FBP images. The noise measurements in the necrotic area were similar to those in muscle, MBIR image reduced noise by 57.8 and 68.9% compared to the 0.625 mm ASIR and FBP images, and was not significant different from the 5 mm FBP images. SNR values for the consolidated lesion of 0.625 mm MBIR image increased by 89.7, 150.0 and 34.1%, compared with the 0.625 mm ASIR, 0.625 mm FBP and 5 mm FBP, respectively (*F* = 43.5, *P* < 0.05), (Table 2). The CNR_(L-C)_ of 0.625 mm MBIR image was increased by 100.0, 23.1 and 166.7%, compared with the 0.625 mm ASIR, 5 mm FBP and 0.625 mm FBP images, (*F* = 27.0, *P* < 0.05), and CNR_(L-A)_ was increased by 343.2, 101.3 and 491.6%, respectively (*F* = 22.9, *P* < 0.05), (Table 3).

**Table 2:** Objective image noise measurements

Tissue	Image^a^	CT value		Noise		SNR	
		Mean ± SD	*P*	Mean ± SD	*P*	Mean ± SD	*P*
Consolidated lesion	MBIR	71.1 ± 9.3	-	13.9 ± 4.0	-	5.5 ± 1.6	-
	ASIR	67.9 ± 12.7	0.13	24.9 ± 6.6	<0.01	2.9 ± 0.9	<0.01
	5 mm FBP	63.9 ± 7.9	<0.01	17.2 ± 6.4	0.02	4.1 ± 1.3	<0.01
	FBP	68.9 ± 12.1	0.10	33.8 ± 8.7	<0.01	2.2 ± 0.6	<0.01
Necrotic area	MBIR	14.8 ± 5.7	-	9.4 ± 1.4	-	1.6 ± 0.6	-
	ASIR	16.2 ± 5.5	0.02	22.3 ± 1.6	<0.01	0.7 ± 0.3	<0.01
	5 mm FBP	15.6 ± 5.0	0.19	9.9 ± 1.8	0.25	1.6 ± 0.6	0.92
	FBP	16.2 ± 5.0	0.05	30.2 ± 3.0	<0.01	0.5 ± 0.2	<0.01
Muscle	MBIR	57.4 ± 7.2	-	12.1 ± 2.2	-	4.9 ± 1.1	-
	ASIR	56.3 ± 6.6	0.48	22.7 ± 4.3	<0.01	2.6 ± 0.7	<0.01
	5 mm FBP	56.6 ± 6.6	0.49	12.9 ± 2.3	0.07	4.5 ± 0.8	0.04
	FBP	56.2 ± 8.9	0.21	30.7 ± 5.8	<0.01	1.9 ± 0.5	<0.01
Air	MBIR	−994.8 ± 4.3	-	3.6 ± 1.9	-	−407.9 ± 280.0	-
	ASIR	−996.7 ± 3.2	0.08	11.2 ± 2.1	<0.01	−92.3 ± 18.8	0.01
	5 mm FBP	−996.8 ± 2.6	0.09	5.2 ± 1.2	<0.01	−203.9 ± 51.4	0.02
	FBP	−996.5 ± 4.0	0.16	15.0 ± 2.7	<0.01	−69.0 ± 14.5	0.01

^a^Images were 0.625 mm in thickness unless stated otherwise

**Table 3:** CNR between model-based iterative reconstruction (MBIR) image and other reconstructions

Algorithm	Consolidated lesion-necrotic area		Consolidated lesion -air	
	CNR	*P*	CNR	*P*
MBIR (0.625 mm)	4.8 ± 1.3	-	436.6 ± 298.5	-
ASIR (0.625 mm)	2.4 ± 1.0	<0.01	98.5 ± 19.8	<0.01
FBP (5 mm)	3.9 ± 1.1	<0.01	216.9 ± 54.7	<0.01
FBP (0.625 mm)	1.8 ± 0.6	<0.01	73.8 ± 15.4	<0.01

### Inter-observer consistency

The inter-observer *Kappa* value for the subjective quality scores was 0.66, showing a good consistency between the two observers.

## Discussion

Necrotizing pneumonia (NP) has no clear definition today. According to Hacimustafaoglu, NP is necrosis in pulmonary consolidation [15], and patchy areas without contrast in a CT scan [16]. Hyewon Seo et al pointed out that NP is distinct form lung abscess, with a characteristic performance of poorly defined foci of low density, and unassociated with enhancing margins in enhanced chest CT [17]. Our own experience indicated that it is very difficult to find necrotic lesions in unenhanced CT, so the standard protocol for NP in our hospital was to perform low dose enhanced CT. NP is currently diagnosed clinically and necrotic lesions are determined in CT images according to experience. Since necrotic lesions have an important significance in the diagnosis of NP, timely and accurate diagnosis is important for guiding future treatment, increasing awareness of complications, follow-up and in providing appropriate recommendations to parents of patients recovering from NP [16]. Previous research on children chest imaging has indicated that MBIR can increase both image quality and image contrast-noise-ratio due to its ability to dramatically decrease image noise and increase spatial resolution [13, 14]. In this study we used the existing standard of care (SOC) low dose CT data. Since SOC low dose CT images meet the clinical diagnostic image quality requirements at 5 mm slice thickness in terms of image noise, by reconstructing the images into 0.625 mm slice thickness, we substantially reduce the signal strength in the thinner slice images to simulate extremely low dose scan conditions while improving the image spatial resolution. The thin-slice (0.625 mm) images were then reconstructed with MBIR, ASIR and conventional FBP algorithms, and the results demonstrated that MBIR algorithm increased image spatial resolution and improved image quality to better delineate necrotic lesions to aid the earlier detection and more confident diagnosis of NP.

Low-dose CT is widely used and necessary, but traditional FBP reconstruction technique provides limited image quality to balance spatial resolution and image noise when using low radiation dose CT scanning, because of the limitation of its mathematical model. More recent noise model-based iterative reconstruction techniques, such as ASIR, use complex statistical models to reduce noise and to produce acceptable image quality even with low-radiation dose [1–5]. The full mode-based iterative reconstruction (MBIR) algorithm further extends the noise reduction capability and at the same time improves the image spatial resolution [8–13] which is not standard on a Discovery CT 750 HD scanner and requires an additional software and hardware. We hypothesized that MBIR can help us to detect even tiny and low contrast necrotic lesions better at their early stage, especially for displaying small necrotic foci of necrosis pneumonia in pediatric patients, even with extremely low radiation doses. But due to paucity of such data, we simulated the extremely low signal situations by using clinically confirmed cases acquired at the standard of care low dosage to validate its capabilities. Since X-ray (signal) detected by the detector is proportional to the thickness of the detection cell, by reconstructing images into the 0.625 mm slice thickness from the standard 5 mm slice thickness, we effectively reduced the X-ray flux in each of the smaller detection cell (0.625 mm vs. 5 mm) by more than 85%, simulating an extremely low signal (low dose) situation. Therefore, the present research was based on the standard-of-care data acquired for clinical purpose. The data acquisition was completed with the conventional scanning procedures for clinical use with no additional operation on patients.

MBIR drastically reduced image noise in such a low signal condition in both lesion and muscle. Our results indicated that the image noise of the 0.625 mm MBIR images was statistically the same as the conventional 5 mm FBP images, and was about 60% lower than that of the 0.625 mm FBP images. In terms of subjective quality, MBIR images were significantly better than FBP and even ASIR images in the overall image quality and visibility of necrotic lesions (Fig. 1). In our study group, there were 33 necrotic lesions observed in 28 pulmonary lobes in the standard 5 mm FBP CT images. The conventional 5 mm images were often too thick to clearly show the boundaries of necrotic lesions, so, not all 33 lesions were confidently determined. The 0.625 mm MBIR images, with higher spatial resolution and much lower image noise, were able to provide clear boundaries for tiny necrotic lesions (Fig. 3). So the 0.625 mm MBIR images not only identified the 33 lesions confidently, but also detected 5 extra lesions (38 vs. 33) in the same 28 lobes. On the other hand, the 0.625 mm FBP images only identified 29 lesions, missing 4 small necrotic lesions from the standard 5 mm FBP images due to the much higher image noise caused by the extremely low signal in the thin slice imaging mode.

In the present study, all included patients were children, wherein the distribution of body fat and length are different from those of adults. Therefore, the association between the radiation dose and BMI was not clear in this population. Consequently, we divided the children into different groups based on their age instead of their body weight or BMI [13]. On the other hand, the body type of children display greater variations with age than adults. In order to obtain a similar proportion of region of interest area in children with different body type, a region with the area about one quarter of the cross-section of the aorta was chosen as the region of interest. Thus, the proportion of the area of the region of interest was the same in different individuals. In addition, the size of the region of interest could be modulated to facilitate the evaluation. Because the necrotic lesions are usually small, it limits the inclusion of a larger area, and a very small range affects the noise result, and hence, we used the well-proportioned back muscle for noise measurement to correct this variation.

This study does have limitations. First, all examinations were performed using 120 kV scan according to the then (before December 2013) standard protocol in our institution. The scan protocols have since been optimized based on patient sizes and clinical tasks. However, we do not expect the tube voltage selection should affect the results of this study significantly, since same data were used with different reconstruction algorithms. Second, because NP is rare in children, the present study was limited by a small sample size. In addition, different noise index used in children with different age could also introduce bias. Further studies with larger sample sizes are necessary to confirm these findings. Third, since the MBIR image quality is much more superior to FBP image, true blinded study was impossible, and there could have been some bias in the subjective quality score. Doctor B provided maximum scores for all evaluation, and this could have possibly affected the fairness of the image quality results to some extent. Third, MBIR is a complex algorithm, it needs quite a long reconstruction time (about 30 min per scan series at the current software version) which also limited the number of cases included in this study and further large-scale research is needed to confirm the conclusion. Finally, we demonstrated the ability of MBIR to dramatically reduce image noise and increase both the high and low contrast resolution compared with the other algorithms in a simulated extreme low signal scanning condition, further research on the performance of MBIR with actual reduced scanning conditions is needed to fully demonstrate it clinical ability.

## Conclusions

The quality of chest CT images in children with necrotizing pneumonia significantly improved by the MBIR technique, especially in the extremely low signal condition, as compared to the FBP reconstruction and noise model-based iterative reconstruction (ASIR), to provide a more confident and accurate diagnosis for necrotizing pneumonia and to help clinicians to develop treatment programs earlier.

## Abbreviations

Air: Background air
ASIR: Adaptive statistical iterative reconstruction
ATCM: Automatic tube current modulation
CNR: Contrast noise ratio
CT: Computed tomography
CTDIvol: CT dose index
DLP: Dose length product
ED: Effective dose
FBP: Filtered back-projection
Le: Consolidated lesion
MBIR: Model-based iterative reconstruction
MPR: Multi-planar reconstruction
Mus: Back muscles
Nec: Necrotic area
NI: Noise index
NP: Necrotizing pneumonia
ROI: Regions of interest
SAFIRE: Sinogram affirmed iterative reconstruction
SD: Standard deviation
SNR: Signal-to-noise ratio
SOC: Standard of care
VR: Volume rendering
